# Whole exome sequencing of suspected mitochondrial patients in clinical practice

**DOI:** 10.1007/s10545-015-9823-y

**Published:** 2015-03-04

**Authors:** Saskia B. Wortmann, David A. Koolen, Jan A. Smeitink, Lambert van den Heuvel, Richard J. Rodenburg

**Affiliations:** 1 Department of Pediatrics, Radboudumc, Nijmegen Centre for Mitochondrial Disorders (NCMD), 774 Translational Metabolic Laboratory, P.O Box 9101, 6500 HB Nijmegen, The Netherlands; 2 Department of Human Genetics, Radboud University Medical Center, Nijmegen, The Netherlands

## Abstract

Mitochondrial disorders are characterized by a broad clinical spectrum. Identical clinical signs and symptoms can be caused by mutations in different mitochondrial or nuclear genes. Vice versa, the same mutation can lead to different phenotypes. Genetic syndromes and neuromuscular disorders mimicking mitochondrial disorders further complicate the diagnostic process. Whole exome sequencing (WES) is the state of the art next generation sequencing technique to identify genetic defects in mitochondrial disorders. Until recently it has mainly been used as a research tool. In this study, the use of WES in routine diagnostics is described. The WES data of 109 patients, referred under the suspicion of a mitochondrial disorder, were examined in two steps. First, the data were filtered using a virtual gene panel of genes known to be associated with mitochondrial disease. If negative, the entire exome was examined. A molecular diagnosis was achieved in 39 % of the heterogeneous cohort, and in 57 % of the subgroup of 42 patients with the highest suspicion for a mitochondrial disease. In addition to mutations in genes known to be associated with mitochondrial disorders (e.g. *TUFM*, *MTFMT*, *FBXL4*), in the subgroup of patients with the lowest suspicion for a mitochondrial disorder we found mutations in several genes associated with neuromuscular disorders (*e.g. SEPN1, ACTA1*) and genetic syndrome (*e.g. SETBP1, ARID1B*). Our results show that WES technology has been successfully implemented as a state-of-the-art, molecular diagnostic test for mitochondrial disorders as well as for the mimicking disorders in daily clinical practice. It also illustrates that clinical and biochemical phenotyping is essential for successful application of WES to diagnose individual patients.

## Introduction

The clinical spectrum of mitochondrial disorders (MD) is extremely broad and the underlying biochemical and genetic defects are heterogeneous (Koopman et al 2012). Identical clinical signs and symptoms can be caused by mutations in different mitochondrial or nuclear genes and, vice versa, the same mutation can lead to different phenotypes. In classical mitochondrial disorders (MD), the primary biochemical defect is located in the oxidative phosphorylation system (OXPHOS). It consists of five multi-subunit enzyme complexes that perform the electron transport of reduction equivalents, generated from the oxidation of mitochondrial substrates, towards molecular oxygen, thereby generating a proton gradient that drives the phosphorylation of ADP into ATP. The genes encoding the subunits of these enzymes are distributed among the mitochondrial DNA (mtDNA) and the nuclear DNA. Mutations in these genes may result in a defect in the corresponding subunit, causing an isolated deficiency of one of the OXPHOS enzymes. In addition, hundreds of different proteins are required for the biosynthesis of the OXPHOS, including assembly factors and mitochondrial translation factors (Mimaki et al 2012; Nouws et al 2012; Smits et al 2010). A defect in one of these proteins often leads to a deficiency of OXPHOS enzymes. Furthermore, a large number of defects outside the OXPHOS have been described that directly or indirectly affect the mitochondrial oxidative ATP production, such as substrate transporters (e.g. *SLC25A3, MPC1*), proteins involved in mitochondrial fission and fusion (e.g. *OPA1, MFN2*), mtDNA maintenance (e.g. *POLG, C10orf2*) or mitochondrial membrane/phospholipid metabolism (e.g. *AGK, SERAC1*). Besides these primary MDs, mitochondrial dysfunction can also be a secondary phenomenon in genetic syndromes and neuromuscular disorders (Katsetos et al 2013; Valenti et al 2014; Wortmann et al 2009). The number of nuclear genes currently known to be associated with MD is more than 250. Novel genetic defects are discovered at high rate due to the rapidly evolving next generation sequencing techniques, such as whole exome sequencing (WES). In the past five years, several papers have been published in which the application of WES to MDs has been reported (Ohtake et al 2014; Taylor et al 2014). Most of this work has been done on a research basis, in particular the studies describing novel genetic defects (Galmiche et al 2011; Haack et al 2014; Yarham et al 2014). In addition to these (mainly case) studies, several papers have described the analysis of selected patient cohorts, for example of patients with an OXPHOS enzyme deficiency in fibroblasts or muscle (Calvo et al 2012; Ohtake et al 2014; Taylor et al 2014). The reported success rates vary between 43 % and 60 %, which is much higher than the 11 % that is achieved by diagnostic Sanger sequencing (Neveling et al 2013). The heterogeneous phenotypical presentation of patients with MD, the heterogeneity of underlying genetic defects, and the large number of proteins involved in the OXPHOS make WES an excellent, state of the art diagnostic tool for mitochondrial disorders. In a proof principle study we have shown that diagnostic application of WES, using a virtual gene panel of 211 genes known to be causative for MD for data filtering, in 44 suspected MD patients had a superior diagnostic yield when compared to standard Sanger sequencing (Neveling et al 2013). Thereafter, our laboratory has been accredited to use WES as a routine diagnostic tool for MD (as well as for a number of other genetic disorders) by the Dutch Accreditation Council. This included setting up standard operating procedures, involvement of clinical geneticists, and procedures for unsolicited findings (Nelen and Veltman 2012; Neveling et al 2013).

Here, we describe the findings in 109 routine diagnostic WES in patients with a suspected MD. The cohort consists of both patients seen in Nijmegen and patients seen elsewhere. Samples from the latter group were sent in by referring physicians under the suspicion of a mitochondrial disease. The clinical suspicion for a mitochondrial disease of the patients varied from relatively low to very high and therefore the cohort represents the heterogeneous group of suspected mitochondrial patients that are investigated in our diagnostic laboratories in daily practice.

## Patients and methods

### Patients

WES was performed in 109 pediatric and young adult patients (up to 27 years of age) under treatment at (or tissues/DNA of patients sent to) the Nijmegen Centre for Mitochondrial Disorders (NCMD) between December 2011 and June 2013. The first 44 patients have also been included in the proof of principle study in which only a mitochondrial disease gene panel was investigated. For these patients, the inclusion criteria were (1) suspicion of a MD by the referring physician, (2) absence of large scale mtDNA deletions and mtDNA point mutations (Iontorrent sequencing or GeneChip® Human Mitochondrial Resequencing Array 2.0 (Affymetrix, Inc); and long template PCR), (3) absence of copy number variations (CNV, 250 k SNP array). For many of the remaining 65 patients, these data were also obtained before proceeding to WES. Written informed consent for WES was obtained after counselling by a clinical geneticist. The patient cohort is representative for the heterogeneous patient population as seen at the NCMD and its referring centers, showing a broad spectrum of clinical signs and symptoms. The most frequently observed features were intellectual disability, developmental delay, myopathy/exercise intolerance, and mitochondrial dysfunction in muscle. It is important to highlight again that WES was performed as a routine diagnostic test and not in a research setting. Retrospectively, the patient cohort was divided into three subgroups. The level of suspicion was defined as “high”, “intermediate” or “low” by thorough (re-)evaluation of the available clinical, metabolic, histological, neuroradiological, and biochemical (measurements of the OXPHOS in fresh muscle and fibroblasts) data of all patients, following the Nijmegen mitochondrial disease scoring system (Morava et al 2006).

*High suspicion of a MD (HS-MD)* includes patients with at least one or more of the following signs and symptoms. Clinical symptoms suggestive of a MD (e.g. chronic progressive external ophthalmoplegia (CPEO), retinitis pigmentosa, optic atrophy, myoclonus epilepsy, polyneuropathy); deficiency of one or more enzymes of the respiratory chain in muscle and cultured fibroblasts; mtDNA depletion in muscle (mtDNA/nDNA ratio below 30 % of mean reference value); ragged red fibres and/or COX-negative fibres in muscle; neuroradiological signs of Leigh syndrome or leukoencephalopathy.

*Intermediate suspicion of a MD (IS-MD)* includes patients with a minimum of two out of three of the following signs and symptoms. Clinical involvement of two or more systems not being intellectual disability/developmental delay in combination with exercise intolerance/excessive fatigue; metabolic evaluation suggestive of a MD (elevated lactate and/or alanine levels in blood and/or cerebrospinal fluid (CSF)); elevated urinary excretion of tricarboxylic acid cycle intermediates or 3-methylglutaconic acid (repeatedly); deficiency of one or more enzymes of the respiratory chain and/or clearly diminished ATP-production from pyruvate/malate in a fresh muscle biopsy.

*Low suspicion of a MD (LS-MD)* includes all patients with intellectual disability (isolated or in combination with exercise intolerance/excessive fatigue without other system involvement), with single system involvement (e.g. isolated cardiomyopathy), or a reduced ATP-production from pyruvate in fresh muscle without additional signs and symptoms suggestive for a MD.

### Molecular genetics and bioinformatics

WES and the data analysis, including the initial filtering step using the virtual MD gene panel, were performed essentially as described before (Neveling et al 2013). An in-house-developed graphical user interface was applied for data visualization and filtering. For clinical interpretation of variants, a routine pipeline was applied to predict the mutation impact at the protein level, which includes data on allele frequency in various databases (dbSNP, EVS (http://evs.gs.washington.edu/EVS), and an in-house database), and the prediction tools SIFT, Polyphen-2 and AlignGVGD (Adzhubei et al 2010; Ng and Henikoff 2001; Tavtigian et al 2006). In addition, genomic conservation as assessed by PhyloP scoring was included in the interpretation of variants (Gilissen et al 2012; Pollard et al 2010). Variants selected in this way, were validated by Sanger sequencing in the probands, and in family members where appropriate.

At the time the data of the patients described here were analysed, the virtual gene panel consisted of 238 genes. These included the genes known to be associated with MDs as well as all other genes encoding the structural subunits of OXPHOS complexes I to V. The panel was designed by a multidisciplinary expert team consisting of a clinical laboratory specialist, a molecular geneticist, and a clinical metabolic specialist. If no mutations were found in the MD gene panel (step 1), the entire exome was investigated (step 2). This included the analysis of a list of genes encoding proteins with a mitochondrial localization, or with a function that is directly linked to mitochondrial function. This list of genes is composed of publically available gene lists, such as MitoCarta (Pagliarini et al 2008), and genes identified in the various research projects in our center. In addition, the exome was investigated for heterozygous mutations in known genes associated with autosomal dominant disorders, or for homozygous/compound heterozygous mutations in all other genes. A clinical report to the referring physician was send after completing step 1 and, if applicable, after step 2.

## Results

The first results of a proof-of-principle study of the implementation of WES as a diagnostic tool at the RadboudUMC Nijmegen in 500 patients, including 44 mitochondrial patients, have been published before (Neveling et al 2013). In the current report, we describe the results of the follow up analysis of these patients (in particular those tested negative for the virtual MD gene panel) and an additional 65 patients (109 patients in total: 52 females, 57 males, aged 1.8 -27.8, median 10.8 years). Biochemical investigations of the OXPHOS in muscle tissue were performed in 92 patients, and in fibroblasts in 94 patients. In muscle tissue of 80 patients, a reduced ATP production from pyruvate + malate oxidation was observed. In 29 patients, a deficiency of a single OXPHOS enzyme was observed, and in 36 patients a combined enzyme deficiency was seen. Single OXPHOS enzyme deficiencies were found in the fibroblasts of six patients, combined enzyme deficiencies in 15 patients. Based on the clinical, metabolic, histological, neuroradiological and biochemical data, 42 patients were assigned to the HS-MD group, 44 to the IS-MD and 23 to the LS-MD group.

A pathogenic mutation explaining the phenotype was found in 42 patients (39 %, see Table 1 for details). The highest diagnostic yield was found in the HS-MD group, in which a genetic diagnosis was made in 24 of 42 patients (57 %). In 16 of these 42 patients (38 %), a pathogenic mutation in one of the genes included in the MD gene panel was found. In another eight patients (19 %) a pathogenic mutation was found outside the MD gene panel. In the IS-MD group of 44 patients, four patients were diagnosed within the MD gene panel (9 %) and another 6 (14 %) outside the MD gene panel. The lowest yield for genetically proven MDs was found in the LS-MD group. In only one of the 23 patients in this group, a causative mutation within the MD gene panel was found. In seven patients (30 %) a genetic diagnosis was detected outside the MD gene panel.

**Table 1:** Overview of the results of the exome sequencing in the patient cohort of in total 109 patients

	Disease causing mutation found in MD gene panel; affected genes	Disease causing mutation found outside MD gene panel; affected genes	Disease causing mutation found cumulative
High suspicion of a mitochondrial disorder HS-MD), N = 42	16/42 (38 %); *COX15* ^c^ *, 2x TK2* ^a,d^ *, SCO2* ^c^ *, c12orf65* ^d^ *, NDUFS1* ^a^ *, NDUFS7* ^a^ *, SURF1* ^c^ *, COA6* ^c^ *, 2x FBXL4* ^d,g^ *, TUFM* ^d^ *, GFM1* ^b^ *, MTFMT* ^d^ *, NDUFV1* ^a^ *, MTO1* ^d^	8/42 (19 %); *SLC3A1/PREPL* ^e^ (cystinuria-hypotonia syndrome, MIM #606407), *2x NGLY1* ^a,e^ (congenital disorder of deglycosylation, MIM #615273), *ARID1B* ^e^ (Coffin-Siris syndrome MIM #135900), *SCN1A* ^a^ (Dravet syndrome, MIM #607208), *unpublished finding 1, 2,3*	24/42 (57 %)
Intermediate suspicion of a mitochondrial disorder (IS-MD), N = 44	4/44 (9 %); *NDUFA1* ^e^ *, RRM2B* ^a^ *, RARS2* ^e^ *, SLC25A4* ^d^	6/44 (14 %); *ASPM* ^e^ *(*autosomal rescessive primary microcephaly5, MIM #608716), *ACTA1* ^a^ (nemaline myopathy3, MIM # 161800), *ALDH4A1* ^e^ (hyperprolinemia II, MIM #606811), *RAPSN* (congenital myasthenic syndrome with actylcholine receptor deficiency, MIM #608931), *COL4A1* (susceptibility to intracerebral hemorrhage, MIM #614519), *unpublished finding 4*	10/44 (23 %)
Low suspicion of a mitochondrial disorder (LS-MD), N = 23	1/23 (4 %); *MFN2* ^e^	7/23 (30 %); *CTNNB* ^f^ mental retardation, autosomal dominant 19, MIM #615075), *KDM6A* ^e^ (Kabuki syndrome 2, MIM#300128), *TBR1* ^e^(MIM *604616, (Palumbo et al 2014)), *SMARCA4* ^f^ (Coffin-Siris syndrome MIM #135900), *SETBP1* ^f^ (Schinzel-Gideon midface retraction syndrome, MIM #269150), *SEPN1* ^e^ (Selenoprotein related myopathy, MIM #602771), *unpublished finding 5*	8/23 (35 %)
All patients included, N = 109	21/109 (19 %)	21/109 (19 %)	42/109 (38 %)

Overview of the results of the exome sequencing in the patient cohort of in total 109 patients. The names of the genes in which mutations have been found are given. The biochemical diagnosis, based on measurements of mitochondrial ATP production rates, and the activity of the enzyme complexes of the oxidative phosphorylation system, is indicated as follows: a) complex I deficiency, b) complex III deficiency, c) complex IV deficiency, d) combined OXPHOS enzyme deficiency, e) reduced ATP production rate but normal OXPHOS enzyme activity, f) normal ATP production and normal OXPHOS enzymes and g) not determined. In all the cases in which an enzyme deficiency has been observed, the ATP production rate was reduced as well

## Discussion

In this study, the use of WES as a routine diagnostic test in 109 patients with a suspected MD has led to a genetic diagnosis in 42 patients (39 %). This is a very satisfactory diagnostic yield when compared to the diagnostic yield achieved by single gene Sanger sequencing (11 %) in a similar patient population, before the introduction of WES (Neveling et al 2013). The diagnostic yield is higher than in previously reported studies in which gene panels have been selectively sequenced. Lieber et al reported a diagnostic yield of 22 % sequencing 1600 genes implicated in mitochondrial biology in 102 patients with suspected MD (Lieber et al 2013). DaRe et al reported a yield of 9.4 % when investigating 447 genes in 148 patients (DaRe et al 2013). The diagnostic yield was the highest in the subgroup of patients with a high suspicion of a MD (57 %, see Table 1). In 21 out of 109 patients (19 %), disease causing mutations were found in one of the genes included in the MD gene panel. In another 21 patients (19 %), pathogenic mutations were found outside the MD gene panel (Table 1). The latter includes mutations in genes causing neuromuscular disorders (*ACTA1* and *SEPN1*), as well as genetic syndromes, such as Kabuki (*KDM6A*), Schinzel Gideon (*SETBP1*) and Coffin-Siris (*ARID1B, SMARCA4*). Several examples of non-MD diagnoses established in this way in patients initially suspected to have a MD have been published before (Davit-Spraul et al 2013; Lieber et al 2012; McCormick et al 2013; Monies et al 2014; Nishri et al 2014). In addition, we identified mutations in genes encoding proteins that are either known to be localized in mitochondria, or have a function associated with mitochondrial energy metabolism in five patients. The details of these findings are outside the scope of the current article, which is to address the feasibility of WES as routine clinical diagnostic tool. Diagnosing patients with clinical features mimicking MD is one of the benefits of using WES as a diagnostic tool. The genetic variants identified by WES were evaluated in relation to the available clinical, metabolic, neuroradiological and biochemical data, by a multidisciplinary team of metabolic pediatricians, clinical geneticists and laboratory specialists. In several cases of our cohort, the diagnosis was only made after a specific sign or symptom of the patient was taken into account; three examples follow:

1. A priori knowledge of the biochemical defect. In the patient with a *GFM1* defect, a heterozygous variant in *GFM1* was present in the WES data. This patient was known to have a combined respiratory chain enzyme deficiency of complex I and IV in fibroblasts. The raw WES data of the *GFM1* gene indicated that part of this gene was not properly covered. Therefore the entire coding region of this gene was sequenced by Sanger sequencing. This led to the identification of a second heterozygous mutation, and to the diagnosis.
2. A priori knowledge of a specific clinical sign. In a patient with microcephaly and mitochondrial dysfunction in muscle no pathogenic mutation was found within the MD gene panel. By examining the entire exome, in particular the many genes known to be associated with microcephaly, led to the finding of mutations in *ASPM*.
3. A priori knowledge of metabolic findings. In a patient with a combined respiratory chain enzyme deficiency in muscle, but not in fibroblasts, no pathogenic mutation was detected within in the MD gene panel. A re-evaluation of the patient, including urinary amino acid profiling, revealed cystinuria. The raw WES data were evaluated for copy number variants in the *SLC3A1* and *PREPL* genes and revealed a homozygous deletion in this region that could be confirmed by Sanger sequencing and led to the diagnosis of hypotonia-cystinuria syndrome. This multi-exonic deletion was not visible in the WES variants list. Another cautionary note in this particular case is that the 250 k SNP array performed prior to WES, failed to identify this micro-deletion/continuous gene syndrome. These examples illustrate that WES enables us to establish a diagnosis in a phenotypically diverse group in which it is often very difficult to discriminate between MD and mimicking disorders. It also underlines that successful application of WES depends on accurate phenotyping.

Currently, the strategies for next generation sequencing of patients with a suspected MD can be divided into two fundamentally different approaches: WES and targeted gene panel sequencing. The pros and cons of these approaches with respect to the application to MD patients have been reviewed recently (Carroll et al 2014). In our opinion, the approach used at the RadboudUMC Nijmegen as described here, combines the advantages of both methods: (1) WES can be used as a generic approach for almost any group of genetic disorders, which is cost-effective as all patients can be examined following the same pipeline. (2) The data can be re-investigated in the future, for example when improved methods for data handling and annotation have been developed, or when “new” genes involved in MD have been discovered. (3) Although WES data interpretation is more complicated and time consuming data in comparison to targeted gene panel sequencing, the use of virtual gene panels to filter the WES data narrows down the number of genetic variants that initially need to be interpreted. (4) If no mutations are detected in the virtual gene panel, the data can be re-evaluated by filtering the data using other gene panels (e.g. neuromuscular disorders) or by a complete exome analysis. This is of special importance given the phenotypic overlap of MD with other metabolic disorders, neuromuscular disorders and genetic syndromes (Briones et al 2001). A disadvantage of WES is that targeted gene panel sequencing gives a higher coverage, although the coverage of WES is continuously being increased. Nevertheless, in the case of a strong suspicion for a mutation in a single or very few genes, targeted sequencing of these candidate genes may be preferred over WES.

Continuous technological innovations have led to reduced costs, shorter turn-around times and increased quality of WES, and it is expected that this trend will continue in the future. As a consequence, WES is increasingly used at an early stage of the diagnostic work-up of suspected mitochondrial patients, before the invasive procedure of a muscle biopsy is considered. However, as our data show, the interpretation of WES data may be compromised by a lack of information on the clinical and biochemical phenotype of the patient. Furthermore, the finding of unknown variants of uncertain pathogenicity in genes not earlier reported to lead to MD will need functional validation in muscle or fibroblasts. Even variants in known disease genes may require functional validation, e.g. in the case of an unusual clinical presentation. This validation may consist of a relatively simple test, such as a Western blot or a specific enzyme assay, e.g. measuring complex I activity. A more reliable confirmation of pathogenicity could be obtained by genetic complementation studies, in which the wild type gene is introduced in cultured patient cells to test if this restores the cellular or biochemical defect. It has been demonstrated before that this strategy is particularly suitable for genetic defects causing mitochondrial disorders (Danhauser et al 2011; Huigsloot et al 2011; Jonckheere et al 2013). Until now, tests like these are being performed in a research setting, but technological innovations may allow for a diagnostic application in the future. As a significant number of MD patients are carriers of pathogenic mtDNA mutations, it is important to note that mtDNA sequencing in clinically affected tissue should be performed prior to WES.

Based on our experiences using WES on patients with varying degrees of suspicion for a MD, we would like to make the following recommendations for the laboratory diagnostic tests to be performed. As described above, WES data analysis benefits from detailed knowledge on the biochemical and clinical phenotype. Therefore, in patients with a high suspicion of a MD, we would advise to start the work up with a muscle and skin biopsy for mitochondrial enzyme measurements and mtDNA sequencing, before performing WES. Should a muscle biopsy for whatever reason not be possible and it is decided to start with WES as a first diagnostic test, we recommend to simultaneously prepare a skin fibroblasts culture for OXPHOS enzyme analysis. This will provide valuable information for the WES data analysis, and the fibroblasts can also be used for the functional validation of genetic variants identified by WES.

Of the genetic defects found outside the MD gene panel, seven genetic defects were found in genes present in the intellectual disability gene panel (ARID1B, SCN1A, ASPM, CTNNB, KDM6A, SMARCA4 and SETBP1). Therefore, in patients with intellectual disability and a medium or low suspicion of a MD, we would advise to perform WES using the virtual gene panels for MD and intellectual disability. The latter panel contains more than 500 genes known to be associated with intellectual disability (de Ligt et al 2012). Additionally a so called TRIO approach including WES analysis of both parents could be considered, allowing the detection of de novo autosomal dominant mutations causing intellectual disability (Vissers et al 2010). Should these approaches not be successful, the next step is likely to be whole genome sequencing, which will be the next step in molecular diagnostic and that has already been shown to have a higher success rate than WES (Gilissen et al 2014).

Taken together, WES technology has been successfully implemented as a routine, state of the art diagnostic test for MD and genetic disorders mimicking MDs. The interpretation of the WES data benefits from knowledge on the clinical, metabolic, neuroradiological and biochemical phenotype, and a thorough evaluation of the data by a multi-disciplinary team.

## References

[CR1] Adzhubei IA, Schmidt S, Peshkin L, Ramensky VE, Gerasimova A, Bork P, Kondrashov AS, Sunyaev SR (2010). A method and server for predicting damaging missense mutations. Nat Methods.

[CR2] Briones P, Vilaseca MA, Garcia-Silva MT, Pineda M, Colomer J, Ferrer I, Artigas J, Jaeken J, Chabas A (2001). Congenital disorders of glycosylation (CDG) may be underdiagnosed when mimicking mitochondrial disease. Eur J Paediatr Neurol.

[CR3] Calvo SE, Compton AG, Hershman SG, Lim SC, Lieber DS, Tucker EJ, Laskowski A, Garone C, Liu S, Jaffe DB, Christodoulou J, Fletcher JM, Bruno DL, Goldblatt J, Dimauro S, Thorburn DR, Mootha VK (2012). Molecular diagnosis of infantile mitochondrial disease with targeted next-generation sequencing. Sci Transl Med.

[CR4] Carroll CJ, Brilhante V, Suomalainen A (2014). Next-generation sequencing for mitochondrial disorders. Br J Pharmacol.

[CR5] Danhauser K, Iuso A, Haack TB, Freisinger P, Brockmann K, Mayr JA, Meitinger T, Prokisch H (2011). Cellular rescue-assay aids verification of causative DNA-variants in mitochondrial complex I deficiency. Mol Genet Metab.

[CR6] DaRe JT, Vasta V, Penn J, Tran NT, Hahn SH (2013). Targeted exome sequencing for mitochondrial disorders reveals high genetic heterogeneity. BMC Med Genet.

[CR7] Davit-Spraul A, Beinat M, Debray D, Rotig A, Slama A, Jacquemin E (2013). Secondary mitochondrial respiratory chain defect can delay accurate PFIC2 diagnosis. JIMD Rep.

[CR8] de Ligt J, Willemsen MH, van Bon BW, Kleefstra T, Yntema HG, Kroes T, Vulto-van Silfhout AT, Koolen DA, de Vries P, Gilissen C, del Rosario M, Hoischen A, Scheffer H, de Vries BB, Brunner HG, Veltman JA, Vissers LE (2012). Diagnostic exome sequencing in persons with severe intellectual disability. N Engl J Med.

[CR9] Galmiche L, Serre V, Beinat M, Assouline Z, Lebre AS, Chretien D, Nietschke P, Benes V, Boddaert N, Sidi D, Brunelle F, Rio M, Munnich A, Rotig A (2011). Exome sequencing identifies MRPL3 mutation in mitochondrial cardiomyopathy. Hum Mutat.

[CR10] Gilissen C, Hehir-Kwa JY, Thung DT, van de Vorst M, van Bon BW, Willemsen MH, Kwint M, Janssen IM, Hoischen A, Schenck A, Leach R, Klein R, Tearle R, Bo T, Pfundt R, Yntema HG, de Vries BB, Kleefstra T, Brunner HG, Vissers LE, Veltman JA (2014). Genome sequencing identifies major causes of severe intellectual disability. Nature.

[CR11] Gilissen C, Hoischen A, Brunner HG, Veltman JA (2012) Disease gene identification strategies for exome sequencing. Eur J Hum Genet 10.1038/ejhg.2011.258 PMC3330229 22258526

[CR12] Haack TB, Gorza M, Danhauser K, Mayr JA, Haberberger B, Wieland T, Kremer L, Strecker V, Graf E, Memari Y, Ahting U, Kopajtich R, Wortmann SB, Rodenburg RJ, Kotzaeridou U, Hoffmann GF, Sperl W, Wittig I, Wilichowski E, Schottmann G, Schuelke M, Plecko B, Stephani U, Strom TM, Meitinger T, Prokisch H, Freisinger P (2014). Phenotypic spectrum of eleven patients and five novel MTFMT mutations identified by exome sequencing and candidate gene screening. Mol Genet Metab.

[CR13] Huigsloot M, Nijtmans LG, Szklarczyk R, Baars MJ, van den Brand MA, Hendriksfranssen MG, van den Heuvel LP, Smeitink JA, Huynen MA, Rodenburg RJ (2011). A mutation in C2orf64 causes impaired cytochrome c oxidase assembly and mitochondrial cardiomyopathy. Am J Hum Genet.

[CR14] Jonckheere AI, Renkema GH, Bras M, van den Heuvel LP, Hoischen A, Gilissen C, Nabuurs SB, Huynen MA, de Vries MC, Smeitink JA, Rodenburg RJ (2013). A complex V ATP5A1 defect causes fatal neonatal mitochondrial encephalopathy. Brain.

[CR15] Katsetos CD, Koutzaki S, Melvin JJ (2013). Mitochondrial dysfunction in neuromuscular disorders. Semin Pediatr Neurol.

[CR16] Koopman WJ, Willems PH, Smeitink JA (2012). Monogenic mitochondrial disorders. N Engl J Med.

[CR17] Lieber DS, Calvo SE, Shanahan K, Slate NG, Liu S, Hershman SG, Gold NB, Chapman BA, Thorburn DR, Berry GT, Schmahmann JD, Borowsky ML, Mueller DM, Sims KB, Mootha VK (2013). Targeted exome sequencing of suspected mitochondrial disorders. Neurology.

[CR18] Lieber DS, Vafai SB, Horton LC, Slate NG, Liu S, Borowsky ML, Calvo SE, Schmahmann JD, Mootha VK (2012). Atypical case of Wolfram syndrome revealed through targeted exome sequencing in a patient with suspected mitochondrial disease. BMC Med Genet.

[CR19] McCormick E, Place E, Falk MJ (2013). Molecular genetic testing for mitochondrial disease: from one generation to the next. Neurotherapeutics.

[CR20] Mimaki M, Wang X, McKenzie M, Thorburn DR, Ryan MT (2012). Understanding mitochondrial complex I assembly in health and disease. Biochim Biophys Acta.

[CR21] Monies DM, Al-Hindi HN, Al-Muhaizea MA, Jaroudi DJ, Al-Younes B, Naim EA, Wakil SM, Meyer BF, Bohlega S (2014). Clinical and pathological heterogeneity of a congenital disorder of glycosylation manifesting as a myasthenic/myopathic syndrome. Neuromuscul Disord.

[CR22] Morava E, van den Heuvel L, Hol F, de Vries MC, Hogeveen M, Rodenburg RJ, Smeitink JA (2006). Mitochondrial disease criteria: diagnostic applications in children. Neurology.

[CR23] Nelen M, Veltman JA (2012). Genome and exome sequencing in the clinic: unbiased genomic approaches with a high diagnostic yield. Pharmacogenomics.

[CR24] Neveling K, Feenstra I, Gilissen C, Hoefsloot LH, Kamsteeg EJ, Mensenkamp AR, Rodenburg RJ, Yntema HG, Spruijt L, Vermeer S, Rinne T, van Gassen KL, Bodmer D, Lugtenberg D, de Reuver R, Buijsman W, Derks RC, Wieskamp N, van den Heuvel B, Ligtenberg MJ, Kremer H, Koolen DA, van de Warrenburg BP, Cremers FP, Marcelis CL, Smeitink JA, Wortmann SB, van Zelst-Stams WA, Veltman JA, Brunner HG, Scheffer H, Nelen MR (2013). A post-hoc comparison of the utility of sanger sequencing and exome sequencing for the diagnosis of heterogeneous diseases. Hum Mutat.

[CR25] Ng PC, Henikoff S (2001). Predicting deleterious amino acid substitutions. Genome Res.

[CR26] Nishri D, Edvardson S, Lev D, Leshinsky-Silver E, Ben-Sira L, Henneke M, Lerman-Sagie T, Blumkin L (2014). Diagnosis by whole exome sequencing of atypical infantile onset Alexander disease masquerading as a mitochondrial disorder. Eur J Paediatr Neurol.

[CR27] Nouws J, Nijtmans LG, Smeitink JA, Vogel RO (2012). Assembly factors as a new class of disease genes for mitochondrial complex I deficiency: cause, pathology and treatment options. Brain.

[CR28] Ohtake A, Murayama K, Mori M, Harashima H, Yamazaki T, Tamaru S, Yamashita Y, Kishita Y, Nakachi Y, Kohda M, Tokuzawa Y, Mizuno Y, Moriyama Y, Kato H, Okazaki Y (2014). Diagnosis and molecular basis of mitochondrial respiratory chain disorders: exome sequencing for disease gene identification. Biochim Biophys Acta.

[CR29] Pagliarini DJ, Calvo SE, Chang B, Sheth SA, Vafai SB, Ong SE, Walford GA, Sugiana C, Boneh A, Chen WK, Hill DE, Vidal M, Evans JG, Thorburn DR, Carr SA, Mootha VK (2008). A mitochondrial protein compendium elucidates complex I disease biology. Cell.

[CR30] Palumbo O, Fichera M, Palumbo P, Rizzo R, Mazzolla E, Cocuzza DM, Carella M, Mattina T (2014). TBR1 is the candidate gene for intellectual disability in patients with a 2q24.2 interstitial deletion. Am J Med Genet A.

[CR31] Pollard KS, Hubisz MJ, Rosenbloom KR, Siepel A (2010). Detection of nonneutral substitution rates on mammalian phylogenies. Genome Res.

[CR32] Smits P, Smeitink J, van den Heuvel L (2010). Mitochondrial translation and beyond: processes implicated in combined oxidative phosphorylation deficiencies. J Biomed Biotechnol.

[CR33] Tavtigian SV, Deffenbaugh AM, Yin L, Judkins T, Scholl T, Samollow PB, de Silva D, Zharkikh A, Thomas A (2006). Comprehensive statistical study of 452 BRCA1 missense substitutions with classification of eight recurrent substitutions as neutral. J Med Genet.

[CR34] Taylor RW, Pyle A, Griffin H, Blakely EL, Duff J, He L, Smertenko T, Alston CL, Neeve VC, Best A, Yarham JW, Kirschner J, Schara U, Talim B, Topaloglu H, Baric I, Holinski-Feder E, Abicht A, Czermin B, Kleinle S, Morris AA, Vassallo G, Gorman GS, Ramesh V, Turnbull D, Santibanez-Koref M, McFarland R, Horvath R, Chinnery PF (2014). Use of whole-exome sequencing to determine the genetic basis of multiple mitochondrial respiratory chain complex deficiencies. JAMA.

[CR35] Valenti D, de Bari L, De Filippis B, Henrion-Caude A, Vacca RA (2014). Mitochondrial dysfunction as a central actor in intellectual disability-related diseases: An overview of Down syndrome, autism. Fragile X and Rett syndrome. Neurosci Biobehav Rev.

[CR36] Vissers LE, de Ligt J, Gilissen C, Janssen I, Steehouwer M, de Vries P, van Lier B, Arts P, Wieskamp N, del Rosario M, van Bon BW, Hoischen A, de Vries BB, Brunner HG, Veltman JA (2010). A de novo paradigm for mental retardation. Nat Genet.

[CR37] Wortmann SB, Zweers-van Essen H, Rodenburg RJ, van den Heuvel LP, de Vries MC, Rasmussen-Conrad E, Smeitink JA, Morava E (2009). Mitochondrial energy production correlates with the age-related BMI. Pediatr Res.

[CR38] Yarham JW, Lamichhane TN, Pyle A, Mattijssen S, Baruffini E, Bruni F, Donnini C, Vassilev A, He L, Blakely EL, Griffin H, Santibanez-Koref M, Bindoff LA, Ferrero I, Chinnery PF, McFarland R, Maraia RJ, Taylor RW (2014). Defective i6A37 modification of mitochondrial and cytosolic tRNAs results from pathogenic mutations in TRIT1 and its substrate tRNA. PLoS Genet.

